# Editorial Brevities

**Published:** 1888-01

**Authors:** 


### EDITORIALS AND MISCELLANEOUS.

### EDITORIAL BREVITIES.

OUR INDEX FOR 1887.—Through the fault of our printers, the index of 1887 was not published in December number, as it should have been. It is bound with the present issue, but in such a way that it can be detached, if desired, and bound with the volume to which it belongs.

TO OUR SUBSCRIBERS.—Bills for amounts due on subscription will be found in the present issue of our journal. We earnestly request those who owe us to pay their subscriptions at once, and continue their journals for 1888. Those who do not notify us to the contrary before the 20th of January, will be regarded as having renewed their subscription. Remember our lIberal offer to extend club rates to every one who sends us a new cash subscriber, to-wit: two copies for $3.

